# Burns Impair Blood-Brain Barrier and Mesenchymal Stem Cells Can Reverse the Process in Mice

**DOI:** 10.3389/fimmu.2020.578879

**Published:** 2020-11-06

**Authors:** Jie Yang, Kui Ma, Cuiping Zhang, Yufan Liu, Feng Liang, Wenzhi Hu, Xiaowei Bian, Siming Yang, Xiaobing Fu

**Affiliations:** ^1^Research Center for Tissue Repair and Regeneration Affiliated to the Medical Innovation Research Department, Chinese People's Liberation Army (PLA) General Hospital and PLA Medical College, Beijing, China; ^2^Department of Dermatology, Fourth Medical Center, PLA General Hospital, Beijing, China; ^3^Tianjin Medical University, Tianjin, China

**Keywords:** burns, blood–brain barrier, mesenchymal stem cells, interleukin-6, interleukin-1β, Mfsd2a

## Abstract

Neurological syndromes are observed in numerous patients who suffer burns, which add to the economic burden of societies and families. Recent studies have implied that blood–brain barrier (BBB) dysfunction is the key factor that induces these central nervous system (CNS) syndromes in peripheral traumatic disease, e.g., surgery and burns. However, the effect of burns on BBB and the underlying mechanism remains, largely, to be determined. The present study aimed to investigate the effect of burns on BBB and the potential of umbilical cord-derived mesenchymal stem cells (UC-MSCs), which have strong anti-inflammatory and repairing ability, to protect the integrity of BBB. BBB permeability was evaluated using dextran tracer (immunohistochemistry imaging and spectrophotometric quantification) and western blot, interleukin (IL)-6, and IL-1β levels in blood and brain were measured by enzyme-linked immunosorbent assay. Furthermore, transmission electron microscopy (TEM) was used to detect transcellular vesicular transport (transcytosis) in BBB. We found that burns increased mouse BBB permeability to both 10-kDa and 70-kDa dextran. IL-6 and IL-1β levels increased in peripheral blood and CNS after burns. In addition, burns decreased the level of tight junction proteins (TJs), including claudin-5, occludin, and ZO-1, which indicated increased BBB permeability due to paracellular pathway. Moreover, increased vesicular density after burns suggested increased transcytosis in brain microvascular endothelial cells. Finally, administering UC-MSCs at 1 h after burns effectively reversed these adverse effects and protected the integrity of BBB. These results suggest that burns increase BBB permeability through both paracellular pathway and transcytosis, the potential mechanism of which might be through increasing IL-6 and IL-1β levels and decreasing Mfsd2a level, and appropriate treatment with UC-MSCs can reverse these effects and protect the integrity of BBB after burns.

## Introduction

Cognitive dysfunction caused by peripheral trauma has been frequently reported, especially postoperative cognitive dysfunction (1–3) and cognitive dysfunction after burns (4–12). These severe central nervous system (CNS) complications burden the patients’ families and the society. Increasing number of studies are focusing on CNS disorders caused by peripheral trauma. Our previous study in mice found that high concentrations of interleukin (IL)-6 in peripheral serum after abdominal surgery increased the permeability of blood–brain barrier (BBB) by disrupting tight junction proteins (TJs), and ultimately led to cognitive dysfunction (1). Danielson et al. found that in patients who underwent open heart surgery, peripheral systemic inflammation caused the dysfunction of the BBB (13). Above all, the pathological process of peripheral trauma that disrupts the integrity of BBB to affect CNS function has been widely accepted in medical field. Compared with the relatively simple incision of surgical trauma, burns cause more severe damage to the skin, elevating the levels of inflammatory factors and harmful substances in the serum. In case of patients with severe burns, serious CNS complications in addition to the damage done to their appearance, may strike the patients and even lead to the onset and progression of more serious psychological disorders. However, whether burns lead to BBB disruption and the underlying mechanisms involved remains to be determined.

BBB is the most important defensive barrier that protect the CNS from poisons and toxins (14–18). Most studies have suggested that its disruption caused by CNS diseases or injuries can exacerbate the development of diseases such as Alzheimer’s disease (19), multiple sclerosis (20), and traumatic brain injury (21). However, few studies have focused on BBB dysfunction caused by peripheral traumatic injury. The dysfunction of BBB leads to peripheral substances infiltrating into the CNS mainly *via* transcellular vesicular transport (transcytosis) and paracellular pathways (22). Our previous study suggested that the peripheral IL-6, a pro-inflammatory cytokine, decrease the level of TJs and thus induce the leakage of BBB to 10-kDa dextran, which may predicted the existence of paracellular pathway after abdominal surgery in mice and using anti-IL-6 neutralizing antibodies could effectively protect the integrity of BBB. However, burn trauma differs from common surgical trauma in two main aspects: First, burn trauma is considered more serious than peripheral surgical trauma, and the factors that induce BBB dysfunction after burns are still unknown. Therefore, use of anti-IL-6 antibodies is not possible. Second, burns belong to one of the most serious types of acute trauma whose occurrence is unpredictable. Therefore, the anti-inflammatory agents, which are limited in their application by the fact that they can be used only a few hours before traumatic injury, such as surgery, cannot be used for burns. Thus, new effective interventions are urgently required.

At present, mesenchymal stem cells (MSCs), which have powerful anti-inflammatory and wound repair effects, have a wide range of applications in medical field (23–26). In addition, some studies have found that MSCs can improve the progression of numerous neurological diseases, such as stroke (27–29), multiple sclerosis (30–32), and Alzheimer’s disease (33–35). Based on these reports, the potential role of MSCs in protecting the integrity of BBB after burn trauma needs to be further studied. MSCs have various strains derived from several tissues including bone marrow, adipose tissue, umbilical cord, placenta, and peripheral blood. Among these, MSCs derived from umbilical cord, also called umbilical cord MSCs (UC-MSCs), have stable biological characteristics, including anti-inflammatory effects and angiogenesis, and are relatively easy to obtain and abundantly available (36–39), making them a perfect choice for the present study. The present study aimed to assess the effect of burns on BBB permeability in animal models. We further aimed to investigate whether UC-MSCs could protect the integrity of BBB after burn trauma.

## Materials and Methods

### Umbilical Cord-Derived Mesenchymal Stem Cell Isolation and Culture

All animal experiments were conducted in accordance with the guidelines of the PLA General Hospital Standing Committee on the Use of Animals in Research and Teaching. One C57BL/6J pregnant mice (about 20 days), purchased from SPF Biotechnology Co., Ltd. (Beijing, China), was employed in this study and eight umbilical cords of which were used to isolate UC-MSCs. Briefly, umbilical cord tissues were harvested from these mice and dissected into small segments in which the veins and arteries were removed; 2 ml type I collagenase (Sigma Aldrich, St. Louis, MO, USA) was added to the tissues that were cultured in 60 mm plates and then were cultured at 37°C in 5% CO_2_ humidified atmosphere for 2 h. And then extracted the cell suspension in plates and placed it in centrifuge (1,000 r * 5 min). The suspension was abandoned and the cells was suspended with Dulbecco’s modified eagle medium (DMEM, Catalog: 2051855, Gibco, Grand Island, NY, USA) with 10% fetal bovine serum (FBS, Gibco, Grand Island, NY, USA) in 100 mm plates (Corning, NY, USA). The umbilical cord explants were cultured at 37°C in 5% CO_2_ humidified atmosphere, and the medium was changed every other day. UC-MSCs phenotype was assessed by flow cytometry based on the positivity for CD29, CD44, and Sca-1 and in the absence of CD31 and CD117 antigens (Cyagen, Santa Clara, CA, USA) (**Figure S1** in **Supplementary Material**). Cells at passage 3 were used for treatment.

### Burn Mice Model and Treatment

All the experiments were performed according to the PLA General Hospital Standing Committee on the Use of Animals in Research and Teaching. Wild-type C57BL/6J female mice, about 8-months-old and weighing about 30 g (SPF Biotechnology Co. Ltd.), were used to generate the burn model. Female mice were used because they were found to be more vulnerable to the disruption of BBB and the development of cognitive impairment after surgery in our previous studies (1, 40). The mice were numbered and randomly assigned to either burn (3, 6, and 12 h) groups, treatment (0, 1, and 3 h) groups, or control group. Mice in both burn and treatment groups were anesthetized with 1% pentobarbital sodium and third-degree scald wounds were inflicted on the back as previously described (41–43). Briefly, a brass probe (diameter = 2 cm) was electrically preheated to 110°C and applied to the dorsal skin of the mice for 10 s. Mice in control group were only anesthetized with 1% pentobarbital sodium. After the procedure, the mice were returned back to their home cages with appropriate food and water and EMLA cream (2.5% lidocaine and 2.5% prilocaine) was applied to the burn site to alleviate the pain (44). Mice in the treatment groups were administered UC-MSCs (0.1 ml suspension containing 1 × 10^6^ cells) at 0, 1, and 3 h after burn trauma, through the tail vein.

### Brain Tissue Harvest

We harvested the brain tissues of the mice for the dextran imaging studies and spectrophotometer quantification of dextran. We harvested the cortex for the Western blot analysis, Enzyme-linked immunosorbent assay and transmission electron microscopy. Each of the mice was perfused with phosphate-buffered saline (PBS) for all assays except dextran imaging studies. After anesthetizing (1% pentobarbital sodium) the mice, thoracotomy was performed and the left ventricle of the heart was perfused slowly with normal saline (NS; five times with 30 ml NS each time) with a needle until the NS overflowing from the right ventricle became colorless. The mice were then decapitated and their brains were harvested. The brain tissues were stored in a −80°C freezer for future analysis as previously described (1).

### Dextran Imaging Studies to Detect Blood–Brain Barrier Permeability

We used dextran to measure BBB permeability as described in previous studies with modifications (1, 22). Briefly, at 3, 6, and 12 h after burns, each mouse was injected with 100 µl 10-kDa dextran tetramethylrhodamine lysine fixable (4 mg/ml, catalog number: D3312, Invitrogen, Carlsbad, CA, USA) and 70-kDa fluoro-Ruby dextran tracer (555/580) (4 mg/ml, catalog number: D1818, Invitrogen, Carlsbad, CA, USA), a high molecular weight tracer, through the tail vein. After 10 min, each mouse was decapitated, brain tissue was harvested, and fixed using 4% paraformaldehyde (PFA) overnight at 4°C. The brain tissues were cryopreserved in 30% sucrose for 1 day and frozen in Tissue-Tek OCT (Sakura). BBB permeability was detected by immunohistochemistry as described previously with modifications (1, 22). The frozen mouse brain hemispheres were cut into 12 µm sections and used for immunohistochemistry staining. These sections were postfixed in 4% PFA at 20–25°C for 15 min, washed in phosphate buffer saline (PBS) three times for 10 min, and permeabilized with 1% Triton X-100 for 10 min. Then, these sections were blocked with 10% albumin from lowlenthal serum for 1 h and incubated with isolectin B4 (1:200; catalog number: I21411, Molecular Probes, San Francisco, CA, USA) overnight at 4°C for immunohistochemistry imaging of blood vessels.

### Spectrophotometric Measurement of Dextran to Detect Blood–Brain Barrier Permeability

Spectrophotometric quantification of the cerebral extracts of the mice was performed as described in previous studies with modifications (22, 45). Briefly, 100 µl dextran tracer [10-kDa fluoro-Ruby dextran tracer (555/580) (4 mg/ml, catalog number: D1817, Invitrogen, Carlsbad, CA, USA) and 70-kDa fluoro-Ruby dextran tracer (555/580) (4 mg/ml, Catalog number: D1818, Invitrogen, Carlsbad, CA, USA)] were injected into the tail vein of each mouse. After 10 min, each mouse was perfused as described above and then decapitated to harvest brain tissues. The amount of 10-kDa and 70-kDa fluoro-Ruby dextran tracer (555/580) in the cerebral extracts was determined by spectrophotometric measurement.

### Transmission Electron Microscopy

At 6 h after burns, mice brains were dissected and fixed by immersion in a 0.1 M sodium-cacodylate-buffered mixture (5% glutaraldehyde and 4% PFA) for 1 h at room temperature followed by 5 h in PFA at 4°C. Following fixation, the tissue was washed overnight in 0.1 M sodium-cacodylate buffer and then cut in 50-µm-thick free-floating sections using a vibrotome. Sections were incubated for 45 min at room temperature in 0.05 M Tris-HCl pH 7.6 buffer, containing 5 mg/10 ml of 3,3′-diaminobenzidine (DAB, Sigma Aldrich, St. Louis, MO, USA) with 0.01% hydrogen peroxide. Sections were then postfixed in 1% osmium tetroxide and 1.5% potassium ferrocyanide, dehydrated, and embedded in epoxy resin. Ultrathin sections (80 nm) were then cut from the block surface, collected on copper grids, stained with Reynold’s lead citrate, and examined under a ht7800 electron microscope.

### Enzyme-Linked Immunosorbent Assay

Mouse IL-6 Immunoassay kit (catalog number: ab100713, Abcam) and the mouse IL-1β Immunoassay kit (catalog number: ab100704, Abcam) were used to determine the levels of IL-6 and IL-1β in blood and cerebral extracts of mice as per the manufacturer’s protocols. Monoclonal antibodies specific for mouse IL-6 and IL-1β were coated onto the microplates. Wells were incubated for 2.5 h at room temperature with test samples (serum) and washed for four times with 300 µl 1× wash buffer. Then, 100 µl mouse IL-6 conjugate was added to each well and incubated for 1 h and the washing step was repeated. Then, 1× HRP-streptavidin was added followed by the washings. Finally, wells were incubated with 100 µl substrate solution for 30 min in the dark and the reaction was stopped with a stop solution (50 µl). Optical density of each well was measured at 450 nm.

### Western Blot Analysis

Western blot analysis was performed as described in our previous study (46). Cerebral tissues were harvested from the control, treatment, and burn groups. Anti-claudin-5 antibody (18-kDa, Catalog number: 352588, 1:1,000 dilution, Invitrogen), anti-occludin antibody (59-kDa, catalog number: ab167161, 1:1,000 dilution, Abcam), anti-ZO-1 antibody (250-kDa, catalog number: ab96587, 1:1,000 dilution, Abcam), aquaporin-4 (AQP-4; 28-kDa, catalog number: ab9512, 1:1,000 dilution, Abcam), matrix metalloprotein-9 (MMP-9; 82-kDa, catalog number: ab38898, 1:1,000 dilution, Abcam), and Mfsd2a (40-kDa, catalog number: ab177881, 1:1,000 dilution, Abcam) were used to detect the level of the proteins in mice brain. Samples from different groups were loaded and β-actin was used to normalize the protein levels (e.g., determining the ratio of claudin-5 to β-actin amount) and as a control for loading differences in the total protein amount.

### Statistical Analyses

Data are presented as mean ± standard deviation (SD). Statistical analysis was performed using GraphPad Prism 8 software. Quantitative data among multi groups’ data were analyzed by one-way analysis of variance (ANOVA). P < 0.05 was considered statistically significant.

## Results

### Burn Increased Extravascular Dextran Level in Mouse Brain

**Figure 1:**
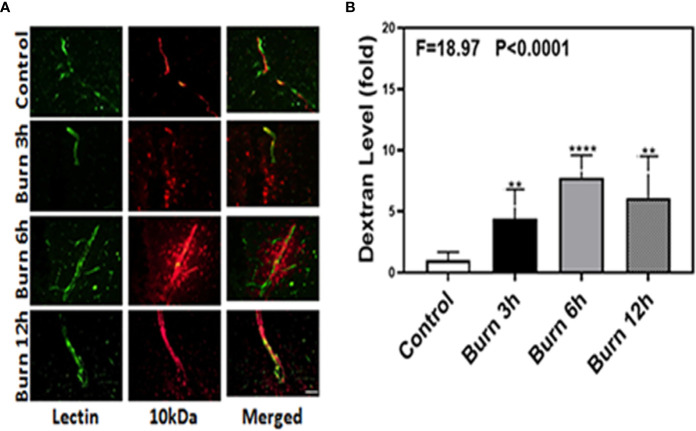
Burn increases blood–brain barrier permeability to 10-kDa dextran in mice. **(A)**. Immunostaining of blood vessels (lectin, green, the first column) and dextran (10-kDa dextran, red, the second column) of the brain section following the control condition in mice (the first row), the burn in mice after 3 h (the second row), the burn in mice after 6 h (the third row), and the burn in mice after 12 h (the fourth row). The third column is the merged image of the first and second columns. The red spots (non-overlap area) in the third column indicate the dextran that is not inside the blood vessel (extravascular dextran). N = total of 90 slides from six mice in each group. Scale bar, 50 µm. **(B)**. Spectrophotometric quantification of brain dextran shows that the burn increases extravascular 10-kDa dextran level in the mouse brain tissues as compared to that in the control condition (white bar)at 3, 6, and 12 h (black, gray, and dot bar) after burn and the highest level is at 6 h (gray bar) after burn. N = 6 in each group. (**p < 0.01, ****p < 0.0001).

We employed immunohistochemistry staining of blood vessels (green color) and 10-kDa dextran (red color) to determine whether burns could increase the BBB permeability in mice at different time points (3, 6, and 12 h). Compared to control condition (**Figure 1A**, first row), burns (**Figure 1A**, second, third, and fourth rows) increased the extravascular 10-kDa dextran levels in the brain tissues of mice at 3, 6, and 12 h after the burn trauma.

**Figure 2:**
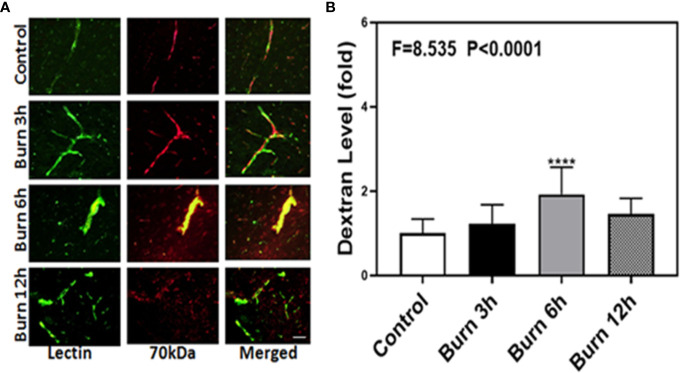
Burn increases blood–brain barrier permeability of 70-kDa dextran in mice. **(A)**. Immunostaining of blood vessels (lectin, green, the first column) and dextran (70-kDa dextran, red, the second column) of the brain section following the control condition in mice (the first row), the burn in mice after 3 h (the second row), the burn in mice after 6 h (the third row), and the burn in mice after 12 h (the fourth row). The third column is the merged image of the first and second columns. The red spots (non-overlap area) in the third column indicate the dextran that is not inside the blood vessel (extravascular dextran). N = total of 90 slides from six mice in each group. Scale bar, 50 µm. **(B)**. Spectrophotometric quantification of brain dextran shows that the burn increases extravascular 70-kDa dextran level in the mouse brain tissues as compared to that in the control condition (white bar) at 3, 6, and 12 h (black, gray, and dot bar) after burn and the highest level is at 6 h (gray bar) after burn. N = 6 in each group. (****p < 0.0001).

We then employed the same method to determine whether burns could increase the BBB permeability to 70-kDa dextran, which has greater molecular weight than 10-kDa dextran, in mice at different time points (3, 6, and 12 h). Compared to control condition (**Figure 2A**, first row), burns (**Figure 2A**, second, third, and fourth rows) increased the extravascular 70-kDa dextran levels in the brain tissues of mice.

Next, we performed spectrophotometric quantification to further confirm that burn trauma could increase the extravascular levels of both 10-kDa and 70-kDa dextran in the brain tissues of mice and found that it indeed increased their levels (**Figures 1** and **2B**, black, gray, and dot bars) compared to that in the control condition (**Figures 1** and **2B**, white bar) (P < 0.0001, one-way ANOVA and Bonferroni test). The highest BBB permeability was detected at 6 h after burns (**Figures 1** and **2B**, gray bar) (P < 0.0001, one-way ANOVA and Bonferroni test). Moreover, the extravascular level of 10-kDa dextran was several times higher than that of 70-kDa dextran after burns. These data suggested that burns were able to increase BBB permeability to both 10-kDa and 70-kDa dextran and the most severe BBB disruption occurred 6 h after burns.

### Umbilical Cord-Derived Mesenchymal Stem Cells Effectively Alleviated Extravascular Dextran Level in Mouse Brain After Burns

**Figure 3:**
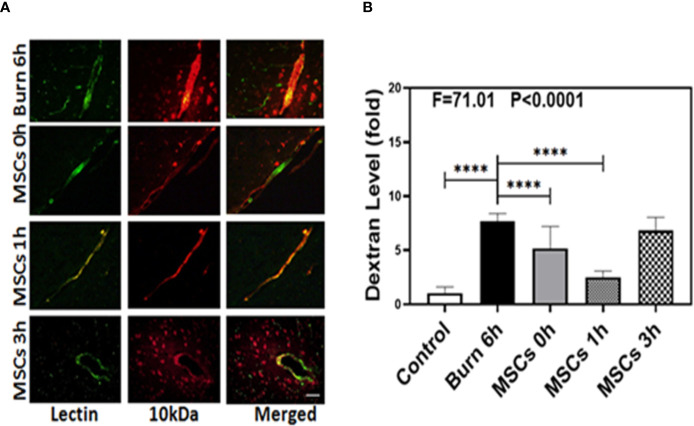
Umbilical cord-derived mesenchymal stem cells (UC-MSCs) alleviate the leakage of blood–brain barrier after burn. **(A)**. Immunostaining of blood vessels (lectin, green, the first column) and dextran (10-kDa dextran, red, the second column) of the brain section following the burn in mice after 6 h (the first row), the UC-MSCs in mice at 0 h after burn (the second row), the UC-MSCs in mice at 1 h after burn (the third row), and the UC-MSCs in mice at 3 h after burn (the fourth row). All mice treated with UC-MSCs were extracted at 6 h after burn. The third column is the merged image of the first and second columns. The red spots (non-overlap area) in the third column indicate the dextran that is not inside the blood vessel (extravascular dextran). N = total of 90 slides from six mice in each group. Scale bar, 50 µm. **(B)**. Spectrophotometric quantification of brain dextran shows that the UC-MSCs decreases extravascular 10-kDa dextran level in the mouse brain tissues as compared to that in the control condition (white bar) at 0 and 1 h (gray and dot bar) after burn and the best result is at 1 h (dot bar) after burn. N = 6 in each group. (****p < 0.0001).

We identified the characteristic and phenotype of UC-MSCs. The flow cytometry analysis showed that sample cells were positive for mesenchymal markers CD29 (96.13%), CD44 (91.74%), and Sca-1 (85.54%) and were negative for CD31 (0.57%) and CD117 (0.80%) (**Figure S1** in **Supplementary Material**). In addition, the microscopic features of sample cells also conform to the morphological characteristics of MSCs (**Figure S2** in **Supplementary Material**). We then employed immunohistochemistry staining of blood vessels (green color) and 10-kDa dextran (red color) to determine whether UC-MSCs treatment (at 0, 1, and 3 h) could improve the integrity of BBB after burns. Compared to the extravascular 10-kDa dextran level at 6 h after burns (**Figure 3A**, first row), the level in the brain tissues of UC-MSCs treated mice (**Figure 3A**, second, third, and fourth rows) was lower. The BBB permeability to extravascular 10-kDa dextran remained a mildly greater increase at 0 and 3 h (**Figure 3A**, second and fourth rows) than 1 h (**Figure 3A**, third row) after using UC-MSCs.

Next, we performed spectrophotometric quantification to further assess whether UC-MSCs treatment (at 0, 1, and 3 h) could effectively attenuate the permeability of BBB after burns. Compared to the control condition (**Figure 3B**, white bar), burns increased the extravascular level of 10-kDa dextran in the brain tissues (**Figure 3B**, black bar; P < 0.0001, one-way ANOVA and Bonferroni test), confirming our previous findings. Moreover, UC-MSC treatment at 0 and 1 h after burns (**Figure 3B**, gray and dot bars) reduced the extravascular 10-kDa dextran level in the brain tissues of mice, compared to that in the burn condition (**Figure 3B**, black bar; P < 0.0001, one-way ANOVA and Bonferroni test). However, UC-MSCs treatment at 3 h after burns (**Figure 3B**, net bar) showed no significant difference in 10-kDa dextran levels compared to that in the burn condition (**Figure 3B**, black bar; P > 0.05, one-way ANOVA and Bonferroni test). The best result was observed in case of UC-MSC treatment at 1 h after burns (**Figure 3B**, dot bar), compared to the treatment at 0 h (**Figure 3B**, gray bar; P < 0.0001, one-way ANOVA and Bonferroni test). These data suggested that UC-MSCs could effectively attenuate the burn-induced BBB permeability and the best time for treatment with UC-MSCs is 1 h after burn trauma.

### Burns Increased IL-1β and IL-6 Levels in Blood and Brain Tissue of Mice

**Figure 4:**
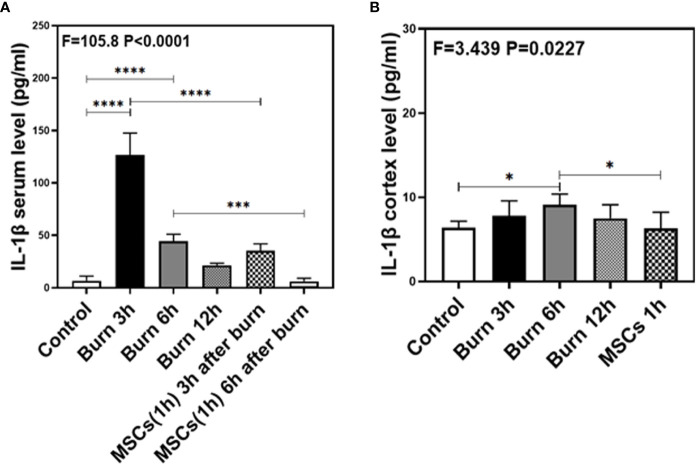
The level of IL-1β in both serum and cortex at different time after burn and the effects of umbilical cord-derived mesenchymal stem cells (UC-MSCs) injected in 1 h after burn on the level of IL-1β. **(A)**. The level of IL-1β at different time and the effects of UC-MSCs (we harvested serum at 3 and 6 h respectively after burn) after burn in serum, N = 3 in each group. **(B)**. The level of IL-1β at different time after burn in brain and the effects of UC-MSCs on the level of IL-1β at 6 h after burn, N = 3 in each group. (*p < 0.05, ***p < 0.001, ****p < 0.0001).

**Figure 5:**
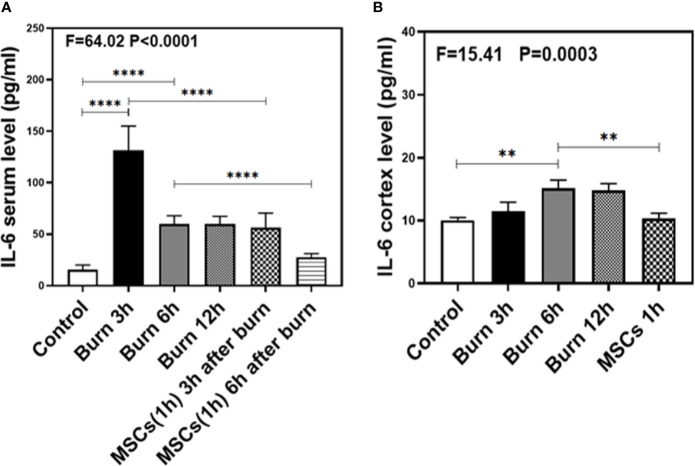
The level of IL-6 in both serum and brain at different time after burn and the effects of umbilical cord-derived mesenchymal stem cells (UC-MSCs) injected in 1 h after burn on the level of IL-6. **(A)**. The level of IL-6 at different time and the effects of UC-MSCs (we harvested serum at 3 and 6 h respectively after burn) after burn in serum, N = 3 in each group. **(B)**. The level of IL-6 at different time after burn in brain and the effects of UC-MSCs on the level of IL-6 at 6 h after burn, N = 3 in each group. (**p < 0.01, ****p < 0.0001).

As our previous research revealed that peripheral inflammatory cytokines induce BBB permeability (1), we investigated the levels of two main pro-inflammatory cytokines, IL-1β and IL-6, in the present study and found them to be elevated in the blood and brain of mice in burn groups. Compared to the control condition (**Figures 4** and **5A**, white bar), IL-1β and IL-6 levels in blood peaked at 3 h after burns (**Figures 4** and **5A**, black bar; P < 0.0001, one-way ANOVA and Bonferroni test), and then decreased slightly at 6 h (**Figures 4** and **5A**, gray bar; P < 0.0001, one-way ANOVA and Bonferroni test) in the burn groups. Further, the IL-1β level decreased to normal at 12 h (**Figure 4A**, dot bar; P = 0.321, one-way ANOVA and Bonferroni test) but the IL-6 level remained slightly increased (**Figure 5A**, dot bar; P = 0.0003, one-way ANOVA and Bonferroni test) in the burn groups. Moreover, compared to the control condition (**Figures 4** and **5B** white bar), IL-1β and IL-6 levels the in brain increased at 6 h after burns (**Figures 4** and **5B**, gray bar; P < 0.05, one-way ANOVA and Bonferroni test), while no significant difference was observed at other time points (**Figures 4** and **5B**, black and dot bars; P > 0.05, one-way ANOVA and Bonferroni test). These data suggested that burns increase the BBB permeability by raising blood IL-1β and IL-6 levels, and these peripheral cytokines infiltrate into the CNS contributing to the CNS inflammation observed in mice.

### Umbilical Cord-Derived Mesenchymal Stem Cells Decreased IL-1β and IL-6 Levels in Blood and Brain Tissue of Mice

Next, we assessed the efficacy of UC-MSCs in decreasing IL-1β and IL-6 levels in the blood and brain of mice after burns. Compared to the burn condition at 3 and 6 h (**Figures 4** and **5A**, black and gray bars), UC-MSCs treated groups showed significantly decreased IL-1β and IL-6 levels in the blood at 3 and 6 h after burns (**Figures 4** and **5A**, net and stripe bars, P < 0.001, one-way ANOVA and Bonferroni test). Moreover, in the mice brain, we found that UC-MSCs treatment significantly decreased both IL-1β and IL-6 levels (**Figures 4** and **5B**, net bar; P < 0.05, one-way ANOVA and Bonferroni test) compared to that in the burn condition after 6 h (**Figures 4** and **5B**, gray bar) and showed no significant difference when compared to that in the control group (**Figures 4** and **5B**, white bar; P > 0.05, one-way ANOVA and Bonferroni test). These data further indicated that peripheral IL-1β and IL-6 were potential key factor in disrupting the BBB after burns and suggested that the possible mechanism about UC-MSCs protecting the integrity of BBB is through decreasing both IL-1β and IL-6 levels in serum, thus attenuating burn-induced neuroinflammation.

### Burns Decreased the Levels of TJs

**Figure 6:**
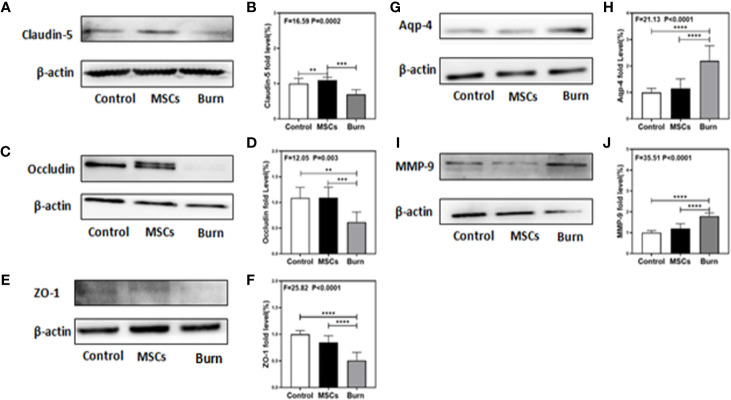
Burn decreases levels of tight junction proteins and increases levels of MMP-9 and AQP-4 while umbilical cord-derived mesenchymal stem cells (UC-MSCs) reversed those processes. UC-MSCs were administered at 1 h after burn and the cortex tissues were extracted at 6 h after burn. **(A, B)**. Burn decreases the claudin-5 levels in the cortex of mice while UC-MSCs increase the levels as compared to the control condition. **(C, D)**. Burn decreases the occludin levels in the cortex of mice while UC-MSCs increase the levels as compared to the control condition. **(E, F)**. Burn decreases the ZO-1 levels in the cortex of mice while UC-MSCs increase the levels as compared to the control condition. **(G, H)**. Burn increases the AQP-4 levels in the cortex of mice while UC-MSCs decrease the levels as compared to the control condition. **(I, J)**. Burn increases the MMP-9 levels in the cortex of mice while UC-MSCs decrease the levels as compared to the control condition. N = 6 in each group (**p < 0.01, ***p < 0.001, ****p < 0.0001).

Our previous study in mice demonstrated the association between peripheral trauma-induced BBB permeability and the alteration of cell junction proteins in cerebral endothelial cells (1). As we found that burns increased BBB permeability to both small and large molecular weight dextran molecules, we evaluated the effect of burns on the levels of TJs, such as claudin-5, occludin, ZO-1, AQP-4, and MMP-9, in the mice brain. Quantitative western blot showed that burns significantly decreased the levels of TJs, claudin-5 (F = 16.59, P = 0.0002, one-way ANOVA; **Figures 6A, B**), occludin (F = 12.05, P = 0.003, one-way ANOVA; **Figures 6C, D**), and ZO-1 (F = 25.82, P < 0.0001, one-way ANOVA; **Figures 6E, F**), after 6 h compared to that in the control condition. Moreover, compared to control condition, burns increased the level of AQP-4 (F = 21.13, P < 0.0001, one-way ANOVA; **Figures 6G, H**), an intrinsic pro-inflammatory protein that is activated only by CNS neuroinflammation and promotes astrocyte swelling, inflammatory cytokine secretion (47), as well as the subsequent occurrence of cerebral edema (28). This result further illustrated that CNS inflammation already existed, which was followed by BBB dysfunction due to burns. Finally, compared to control condition, burns increased the level of MMP-9 (F = 35.51, P < 0.0001, one-way ANOVA; **Figures 6I, J**), a main protease disrupting the TJs and basal membrane of brain microvascular endothelial cells, which could be increased with the increasing level of IL-6 (3, 48, 49). Above all, these data suggested that burns induce the BBB dysfunction by decreasing the levels of TJs and increasing the level of MMP-9, thereby inducing neuroinflammation.

### Umbilical Cord-Derived Mesenchymal Stem Cells Attenuated the Decrease in Levels of TJs

As we found that burns decreased the levels of TJs, we assessed the efficacy of UC-MSCs in attenuating this decrease. Quantitative western blot showed that UC-MSC treatment significantly attenuated the decreasing the levels of TJs, claudin-5 (F = 16.59, P = 0.0002, one-way ANOVA; **Figures 6A, B**), occludin (F = 12.05, P = 0.003, one-way ANOVA; **Figures 6C, D**), and ZO-1 (F = 25.82, P < 0.0001, one-way ANOVA; **Figures 6E, F**), compared to burn condition after 6 h. Moreover, UC-MSCs attenuated the increasing level of AQP-4 (F = 21.13, P < 0.0001, one-way ANOVA; **Figures 6G, H**) and MMP-9 (F = 35.51, P < 0.0001, one-way ANOVA; **Figures 6I, J**) compared to that in the burn condition after 6 h. These data suggested that UC-MSCs prevented the impairment of BBB by attenuating the decreased levels of TJs and the increased level of MMP-9, and thus the neuroinflammation.

### Burns Increased Transcytosis in Cerebral Endothelial Cells

**Figure 7:**
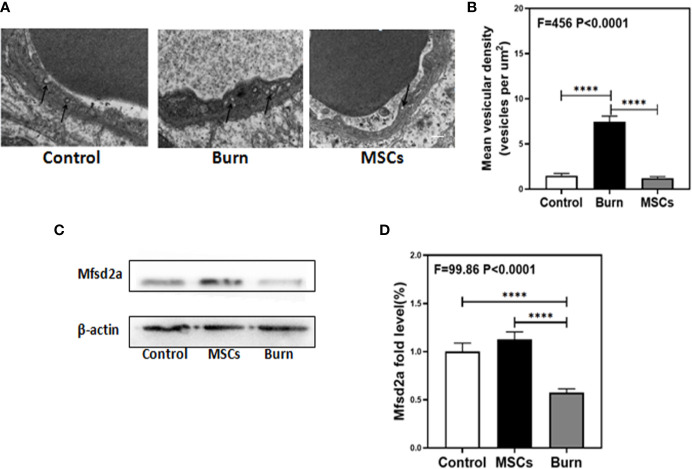
Burn increases transcytosis in brain endothelium while umbilical cord-derived mesenchymal stem cells (UC-MSCs) suppressed the transcytosis process. UC-MSCs were administered at 1 h after burn and the cortex tissues were extracted at 6 h after burn in each group. **(A, B)**. Electron-microscopy examination of BBB integrity. Burn increases vesicular activity in brain endothelium while UC-MSCs decrease the activity as compared to the control. N=3 in each group. **(C, D)**. Western blot. Burn decreases the Mfsd2a levels in the cortex of mice while UC-MSCs increase the levels as compared to the control condition. Scale bar, 200nm. N = 6 in each group (****p < 0.0001).

The decreased levels of TJs in burns group indicated the involvement of paracellular pathway in BBB; therefore, we investigated the involvement of other pathways. Transmission electron microscopy (TEM) showed that compared to the control condition (**Figure 7A, B**, white bar), burns increased the number of vesicles in the BBB (**Figure 7A, B**, black bar; P < 0.0001, one-way ANOVA and Bonferroni test), which indicate that transcytosis might increase across BBB after burn trauma. Western blot showed that compared to the control condition (**Figure 7C, D** white bar), burns decreased the level of Mfsd2a (**Figure 7C, D**, gray bar; P < 0.0001, one-way ANOVA and Bonferroni test). Mfsd2a is a protein that is closely associated with transcytosis. The decrease in Mfsd2a levels induces an increase in transcytosis across the BBB (22). These data suggested that burns might induce and enhance transcellular effects by decreasing the protein level of Mfsd2a.

### Umbilical Cord-Derived Mesenchymal Stem Cells Inhibited Transcytosis in Cerebral Endothelial Cells

Next, we determined whether UC-MSCs could further inhibit the transcytosis in cerebral endothelial cells. TEM showed that compared to the burn condition (**Figure 7A, B**, black bar), UC-MSCs prevented an increase in the number of vesicles in BBB (**Figure 7A, B**, gray bar; P < 0.0001, one-way ANOVA and Bonferroni test). Further, Western blot showed that compared to the burn condition (**Figure 7C, D** gray bar), UC-MSC treatment increased the level of Mfsd2a (**Figure 7C, D**, black bar; P < 0.0001, one-way ANOVA and Bonferroni test).These data suggested that UC-MSCs may inhibit transcytosis by increasing the level of Mfsd2a.

## Discussion

Burns have been shown to induce cognitive impairment and abnormal behavior (4, 8–12). Given the fact that BBB dysfunction is the key factor in cognitive impairment (50–57) and that MSCs can effectively improve the integrity of BBB, we investigated whether burns could induce BBB dysfunction and neuroinflammation as well as evaluated its potential mechanism and the best time point for intervention to improve these effects after burn.

The sex of mice we have chosen for burn model was female. Although burn injury seem to be disproportionately an injury that occurs in males, female may be more vulnerable to the development of cognitive impairment after burn. Wasiak et al. found that in the 12 months post-injury, female patients showed overall poorer physical and mental health status, greater psychological distress, and greater difficulty with aspects of burn-specific health-related quality of life (HRQoL) (58). Other researches show that female after burn is more vulnerable to worse scar quality and mortality (59, 60). Therefore, we only used female mice in the current studies. Furthermore, because dextran-tracer is usually used to determine BBB integrity (61–68), we used it to estimate the effect of burn on BBB integrity in our research. We found that that burns increased the BBB permeability to 10-kDa as well as 70-kDa dextran, which is more difficult to infiltrate into CNS owing to its larger size. In addition, other studies have already demonstrated that the dosing of UC-MSCs (1 × 10^6^) we have chosen is effective and safe. Pati et al. (69) have administered MSCs (1 × 10^6^) for transplantation *via* tail vein at 2 and 24 h after traumatic brain injury in mice. Menge et al. (70) have administered MSCs (1 × 10^6^) to transplant *via* tail vein at 2 and 24 h after traumatic brain injury in mice as well. Liu et al. (71) have administered MSCs (5 × 10^5^) to transplant *via* tail vein at disease onset in EAE mice. Our current study has found that injecting UC-MSCs through tail veins of the mice at 0, 1, and 3 h after burn attenuated the burn-induced increase in BBB permeability. This suggested that UC-MSC treatment at 1 h after burn trauma could effectively improve the BBB permeability.

IL-6 and IL-1β are the main inflammatory cytokines that are released during inflammation, and their uncontrolled secretion can either induce or deteriorate the onset and progression of various diseases (1, 72–74). In addition, recent research found the overexpression of both cytokines was associated with the disruption of BBB. Uchida et al. (75) found that increasing IL-6 level in cerebrospinal fluid (CSF) of patients with neuromyelitis optica led to the disruption of BBB. Our previous research findings also confirmed that increasing IL-6 level in peripheral blood after abdominal surgery induced an increase in BBB permeability (1). Therefore, to investigate the effects of these inflammatory cytokines in the BBB after burns, we detected their levels in both serum and brain at different time points after burn trauma in mice. Our findings showed that the levels of IL-6 and IL-1β peaked at 3 h but decreased at 6 and 12 h after burns in serum. Interestingly, the levels of both inflammatory cytokines were increased at 6 h after burn in the brain, which indicated that burn-induced peripheral IL-6 and IL-1β may be the key factors inducing BBB dysfunction and could eventually lead to neuroinflammation. Moreover, these cytokines were found to be neutralized by the injection of UC-MSCs at 1 h after burn trauma. These data supported the hypothesis that the burn might induce an IL-1β- and IL-6-dependent increase in BBB permeability and neuroinflammation in mice and that UC-MSC treatment within 1 h after burn trauma could effectively protect the integrity of BBB and prevent infiltration of inflammatory cytokines including IL-6 and IL-1β.

Western blot analyses showed that the increasing BBB permeability after burn trauma was induced by the decrease in TJs levels and increase in MMP-9 level, which indicated that the burn-induced dysfunction of BBB involved increasing the paracellular pathway. The double bands seen for occludin in the Western blot analyses might be resulted from different structure of the proteins in tissues or the existence of non-specific proteins. Additionally, TEM showed that burns increased the number of vesicles in BBB, which indicated the involvement of transcytosis in increasing the BBB permeability as well. Recent research has confirmed that Mfsd2a deficiency could induce an increase in transcytosis, and compensatory increase in its expression could restore the low permeability of BBB (22, 76). Based on this, our findings suggest that the transcytosis of BBB increased by decreasing the protein level of Mfsd2a after burn trauma. Moreover, UC-MSCs that were injected at 1 h after burn trauma protected TJs, including claudin-5, occludin, and ZO-1, prevented MMP-9 increase, and inhibited transcytosis by enhancing the expression of Mfsd2a, thus recovering the integrity of BBB.

Recent studies illustrated the function of AQP-4 as a water and solute clearance system that is regulated by astrocytes (77). Its normal biological function is to facilitate the removal of waste from the brain (78). Moreover, AQP-4, an intrinsic pro-inflammatory factor, is only activated by the inflammatory factors in the brain tissue and promotes swelling in astrocytes, secretion of inflammatory cytokines (47, 79), as well as the occurrence of cerebral edema (28, 80). In the present study, we detect increased level of AQP-4 in mouse brain after burns, which further confirmed that burns could induce neuroinflammation, mainly *via* the infiltration of peripheral cytokines. Further, UC-MSCs treatment at 1 h after burns effectively inhibited the increase in AQP-4 level. These results suggest two hypotheses: first, UC-MSCs inhibit the increasing level of AQP-4 by inhibiting the burn-induced neuroinflammation. Second, UC-MSCs directly inhibit the increasing level of AQP-4. However, the underlying mechanism need to be further studied.

Our data collectively indicate that burn trauma induced the disruption of BBB, which is most critical after 6 h. We further identified the potential mechanism by which burns initially increase the BBB permeability: Burns increase the levels of peripheral inflammatory cytokines, including IL-1β and IL-6, which then infiltrate into the CNS by disrupting the BBB and thus induce the neuroinflammation. Li et al. reported that AQP-4, being an intrinsic pro-inflammatory factor, could be activated by the intrinsic neuroinflammation alone (47). Combined with our results, we inferred that the infiltrating cytokines would further activate the expression of AQP-4 and thus exacerbate the neuroinflammation. Our results have already confirmed that increased permeability of BBB after burns was caused by the disruption of TJs, paracellular pathway, decreased Mfsd2a level, and transcytosis. However, the potential mechanism of Mfsd2a decrease after burn trauma needs further investigation. Finally, UC-MSCs inhibited the overexpression of IL-6 and IL-1β, protected the TJs, and impeded the infiltration of pro-inflammatory cytokines into the CNS, thus contributing to the attenuation of CNS neuroinflammation. However, while the potential effects of UC-MSCs on AQP-4 and the underlying mechanisms remain unknown, the decreasing level of AQP-4 in UC-MSCs treated mice further confirmed their efficacy in attenuating the CNS inflammation.

In conclusion, our data suggested that burns could induce an increase in the permeability of BBB *via* paracellular pathway as well as transcytosis. Specifically, as burns increased the levels of both IL-6 and IL-1β in mice, we inferred that they may affect the permeability of BBB in two ways: First, the increased levels of IL-6 and IL- β observed in the present study induced the disruption of TJs, including claudin-5, occludin and ZO-1, which facilitated the infiltration of peripheral substances into the CNS *via* paracellular pathway. Second, burns increased the transcytosis in BBB by decreasing the protein level of Mfsd2a, but the underlying mechanism remains to be determined. Moreover, both ways would eventually lead to the CNS inflammation. Subsequently, these blood cytokines would infiltrate into the CNS *via* either of the two pathways to further induce increase in AQP-4 level, and thus aggravate the neuroinflammation. Finally, we demonstrated that UC-MSCs injected through tail vein in mice at 1 h after burn trauma effectively reversed these adverse effects and thus protect the integrity of BBB. Above all, the present study established a system to study the effects of burn and the injection of UC-MSCs on BBB permeability in mice and investigate the best treatment time point. With fast-paced modern life, the incidences of traumatic injury have increased worldwide, and the complications in CNS induced by peripheral traumatic incidences should be considered. Those who are suffering from such injuries or diseases will be likely afflicted with acute trauma-induced neuroinflammation, which will finally lead to neurological degeneration (81–83). To avoid such eventuality in these cases, it is important to protect the integrity of the key gate—the BBB. Our current research provided a new approach to protect the integrity of BBB from disruption. Administrating UC-MSCs after burn trauma could effectively improve the BBB permeability; thus, we provide evidence as well as basis for translation to the clinic, and further studies are required for evaluating this treatment method.

## Data Availability Statement

The raw data supporting the conclusions of this article will be made available by the authors, without undue reservation.

## Ethics Statement

The animal study was reviewed and approved by PLA General Hospital Standing Committee on the Use of Animals in Research and Teaching.

## Author Contributions

JY, SY, CZ, and XF conceived and designed the project. JY, KM, YL, FL, WH, XB, and SY performed all the experiments and prepared the figures. JY, SY, and XF wrote the manuscript. All authors contributed to the article and approved the submitted version.

## Funding

This study was supported in part by the National Nature Science Foundation of China (81801909, 81830064, 81721092), Beijing Natural Science Foundation (7184245), the National Key Research and Development Plan (2017YFC1103304), and the Military Medical Research and Development Projects (18-JCJQ-QT-020, AWS17J005).

## Conflict of Interest

The authors declare that the research was conducted in the absence of any commercial or financial relationships that could be construed as a potential conflict of interest.
