# Supplementary material for: Burns Impair Blood-Brain Barrier and Mesenchymal Stem Cells Can Reverse the Process in Mice
Source: Front Immunol. 2020 Nov 6;11:578879. doi: 10.3389/fimmu.2020.578879 (PMC7677525; doi:10.3389/fimmu.2020.578879)
Supplement: Supplementary file 1 [file Image_1.pdf]

## Supplementary Material

## S-Figure1

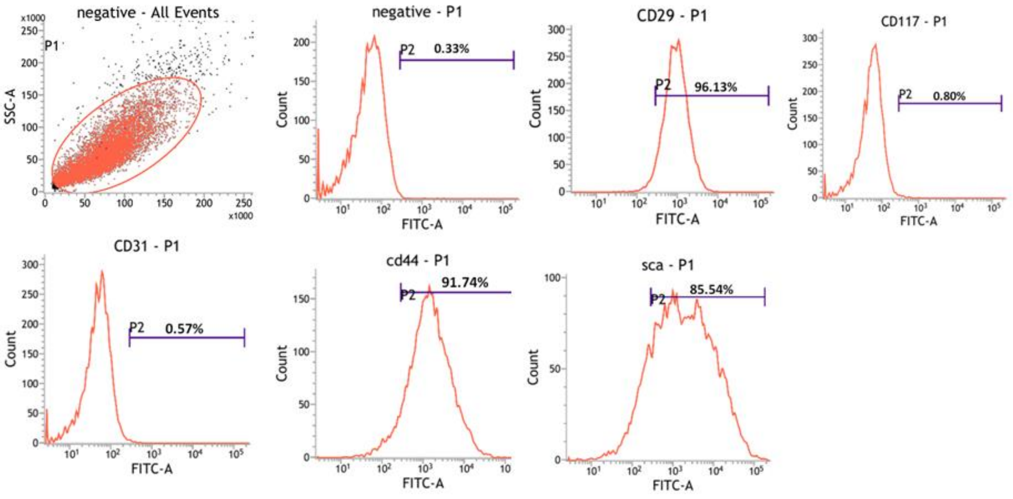


**S-Figure 1.** The flow cytometry analysis of UC-MSCs. The positive mesenchymal markers include CD29 CD44 and Sca-1, the negative markers include CD31 and CD117.

## S-Figure2

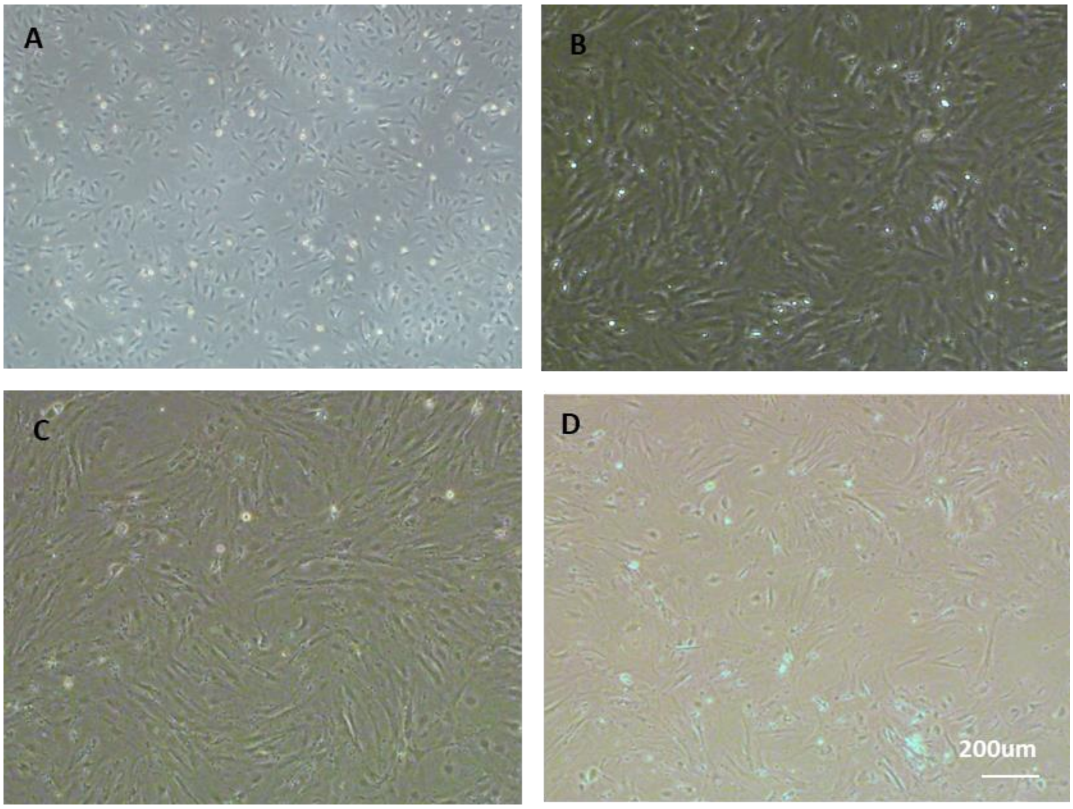

**S-Figure 2.** The shape of UC-MSCs. (A).The shape of primary mesenchymal stem cells. (B).The shape of passage one mesenchymal stem cells. (C).The shape of passage two mesenchymal stem cells. (D).The shape of passage three mesenchymal stem cells.
